# Parental Decision‐Making and Pregnancy Outcomes After Increased First‐Trimester Nuchal Translucency: A 12‐Year Cohort

**DOI:** 10.1002/pd.70224

**Published:** 2026-07-14

**Authors:** Benjamin Birene, Jean‐Paul Bory, Eloi Dondeyne, Anne Charbonneau, Clémence Jacquin, Emilie Landais, Mohamed Dakin, Olivier Graesslin

**Affiliations:** ^1^ Obstetrics and Gynecology Department Centre Hospitalier Universitaire de Reims Reims France; ^2^ Paediatric and Congenital Cardiology Unit Centre Hospitalier Universitaire de Reims Reims France; ^3^ Genetics Department Centre Hospitalier Universitaire de Reims Reims France

**Keywords:** chromosome disorders, diagnosis, fetal diseases, nuchal translucency measurement, parents, psychology

## Abstract

**Objective:**

To describe diagnostic trajectories and parental decision‐making following increased first‐trimester nuchal translucency (NT), according to NT thickness.

**Method:**

This 12‐year retrospective cohort study was conducted at a French tertiary Prenatal Diagnosis and Fetal Medicine Center and included 316 singleton pregnancies with first‐trimester NT ≥ 3.5 mm, with or without structural anomalies.

**Results:**

Termination of pregnancy occurred in 180/316 pregnancies (57.0%), while 91/316 (28.8%) resulted in live birth without pathology. TOP without prior invasive prenatal testing increased from 7.4% in the 3.5–< 5.0 mm group to 35.0% in the ≥ 6.5 mm group. Invasive prenatal testing was performed in 248 pregnancies. Among 137 pregnancies with an abnormal genetic result and known outcome, 12 (8.8%) were continued. Structural ultrasound assessment was performed in 111 pregnancies; 17/29 (58.6%) with abnormal findings were continued. Among 51 pregnancies that continued after normal genetic testing and normal structural ultrasound, 42 (82.4%) resulted in live birth without pathology at the last follow‐up, including 3/8 (37.5%) with NT ≥ 6.5 mm.

**Conclusion:**

Parental decisions and outcomes varied with NT thickness. Integrating NT measurements with sequential genetic and ultrasound findings may support individualized prenatal counseling.

## Summary

- What is already known about this topic?
  ◦ Increased first‐trimester NT is associated with chromosomal, genetic, and structural abnormalities, with risks increasing alongside NT thickness.
- What does this study add?
  ◦ Parental decisions varied with NT thickness, with more frequent termination before invasive testing and lower continuation rates after invasive testing as NT increased.
  ◦ After normal genetic testing and structural ultrasound, most continued pregnancies resulted in live birth without detected pathology, although this proportion was lower when NT was ≥ 6.5 mm.

## 1 Introduction

Increased nuchal translucency (NT), commonly defined as a measurement exceeding 3.5 mm or above the 95th percentile between 11 and 14 weeks of gestation, is a well‐established early marker for a broad spectrum of fetal anomalies [1, 2]. The prevalence of increased NT is estimated to be around 0.5% of pregnancies [3]. Although increased NT can occasionally be an isolated and transient finding, its presence is frequently associated with chromosomal abnormalities, genetic syndromes, structural malformations, and, more broadly, adverse perinatal outcomes [4].

The detection of increased NT typically initiates a complex diagnostic pathway involving first‐ and second‐tier genetic testing, detailed fetal imaging, and multidisciplinary consultations. These investigations often result in emotionally and ethically complex decisions, including whether to continue the pregnancy [5]. While the diagnostic yield of increased NT has been extensively described in the literature, particularly with the increasing use of chromosomal microarray and exome sequencing [6, 7], the real‐world impact of these findings on the subsequent course of care and parental decision‐making remains less thoroughly explored.

A more comprehensive understanding of these trajectories is essential to better support families in early pregnancy and to optimize prenatal counseling.

The aim of this study was to describe the diagnostic trajectories, care pathways, and parental decision‐making in pregnancies identified with increased first‐trimester nuchal translucency within a tertiary referral center, with particular attention to variations according to NT thickness, influence of genetic and structural ultrasound findings on pregnancy continuation, documented indications for termination of pregnancy, and outcomes after reassuring prenatal investigations.

## 2 Material and Methods

### 2.1 Sample

This was a single‐center retrospective cohort study carried out at the Prenatal Diagnosis and Fetal Medicine Center (CPDPN) in Reims over a 12‐year period (from January 1, 2013, to December 31, 2024). In France, a CPDPN is a facility that brings together a multidisciplinary team of professionals to optimize prenatal diagnostic procedures, provide information regarding therapeutic options and prognosis, coordinate pregnancy management, and plan for care of the unborn child. It is also entrusted with certifying the particular severity and incurability of certain fetal disorders that may justify a Termination of Pregnancy (TOP) [8].

Cases eligible for this study were identified from summary tables submitted to the Biomedicine Agency and included all those who presented with a first‐trimester NT of at least 3.5 mm between 11 and 14 weeks of gestation, irrespective of whether it was described as a cystic hygroma or increased NT, with or without additional structural anomalies. Exclusion criteria were the absence of a high‐quality first‐trimester ultrasound and multiple pregnancies. Cases with TOP, live births, and intrauterine fetal demise were all included. Data were extracted from medical records, including prenatal consultations, genetic testing results, ultrasound reports, delivery records, and neonatal charts.

### 2.2 Data

We collected data on maternal demographics, pregnancy characteristics, and prenatal management. This included gestational age and type of invasive genetic testing (chorionic villus sampling or amniocentesis) as well as the timing of termination of pregnancy or stillbirth, and the most recent ultrasound or genetic assessment prior to these outcomes. Morphological ultrasound findings were categorized by system and severity. Genetic investigations included non‐invasive prenatal testing (NIPT), fluorescence in situ hybridization (FISH), conventional chromosome analysis, chromosomal microarray analysis (CMA), and, when indicated, exome sequencing or postnatal genetic analysis. Genetic etiologies were recorded and classified according to the technique used for diagnosis. We also analyzed the motivations for requesting TOP, which were categorized as follows: abnormal genetic test results, abnormal ultrasound findings, concerns regarding long‐term prognosis, other reasons, or missing data. All cases with a known pregnancy outcome were included in the final analysis.

Pregnancy outcomes were classified into the following categories: termination of pregnancy, stillbirth, neonatal death, live birth with pathology, or live birth without pathology. The classification “live birth with pathology” encompassed any diagnosed condition, irrespective of its severity or curability.

Nuchal translucency was assessed using Nicolaides' curves [9]. NT thickness was stratified into three groups (3.5–< 5 mm, 5–< 6.5 mm, and ≥ 6.5 mm), consistent with the clinical thresholds applied in routine management at our center. A first‐trimester ultrasound was considered high‐quality if performed between 11 and 14 weeks of gestation with a Herman score of at least 7 [10, 11]. For pathological outcomes, only diagnoses confirmed by pathology or genetic testing were retained. All sonographers involved in NT measurements undergo annual certification and quality assessment. Structural ultrasound assessment included examinations performed at approximately 18 weeks of gestation or later; first‐trimester ultrasound findings were excluded. Only genetic and ultrasound findings available before the parental decision were considered.

Because of the retrospective design of the study, individual informed consent was not required. After receiving appropriate information, no patient opposed the use of their medical data for research. The authors state that they have adhered to the ethical principles outlined in the Declaration of Helsinki. This study was approved by our local ethics committee (IRB No. 2024‐OBS‐1209) [12].

### 2.3 Statistical Analysis

Descriptive statistics were used to summarize the data. Categorical variables are presented as numbers and percentages, and continuous variables as means ± standard deviations or medians with interquartile ranges, depending on distribution. Percentages were calculated using the number of pregnancies eligible for each specific analysis as the denominator; cases with missing relevant data were excluded from the corresponding analysis. All statistical analyses were performed using SAS 9.4 (SAS Institute, Cary, NC). No formal sample size calculation was performed, as this study included all consecutive eligible pregnancies managed at our center over the 12‐year study period.

## 3 Results

**FIGURE 1:**
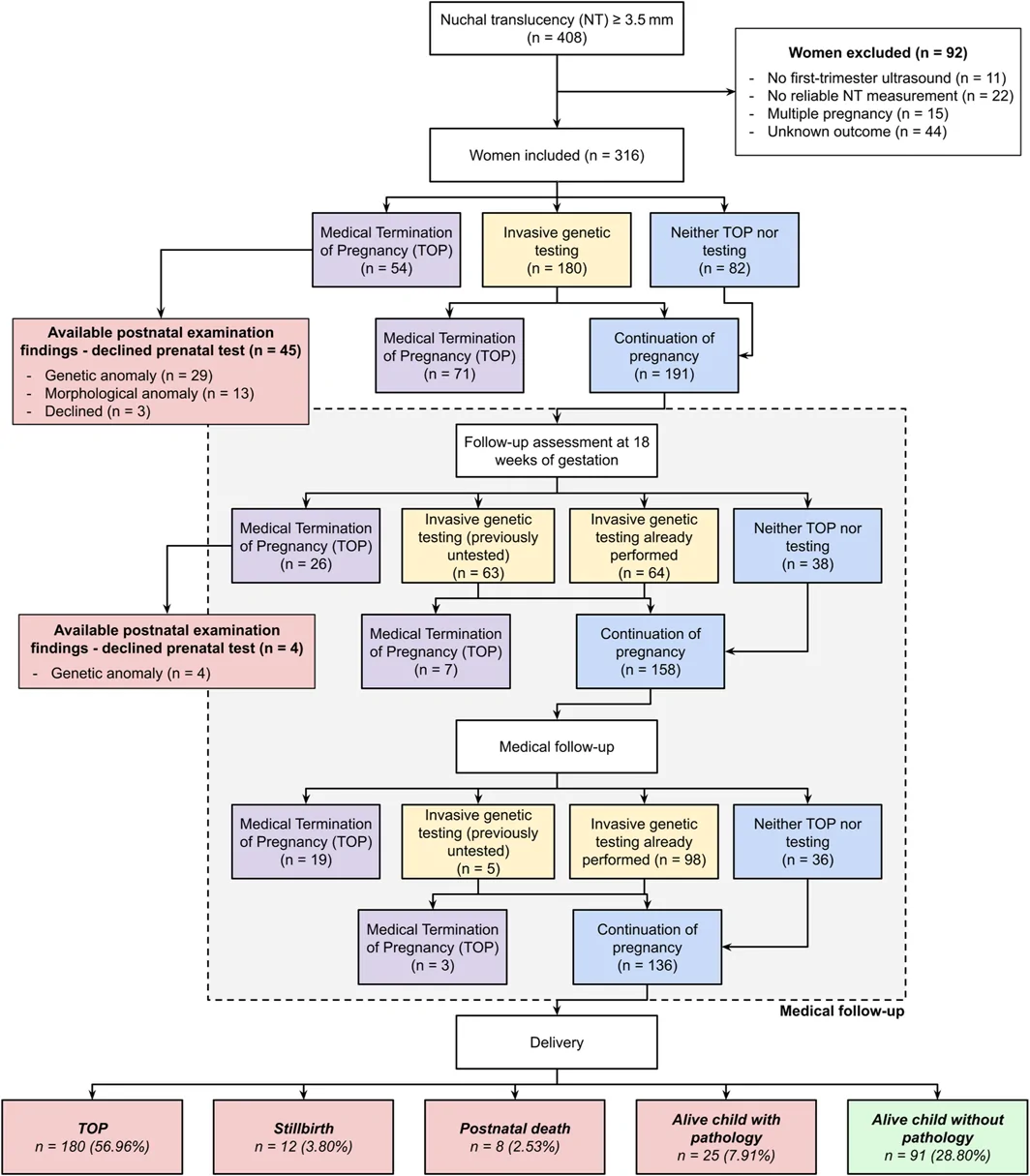
Study flowchart.

A total of 316 pregnancies were included in the study. Among them, 148 (46.8%) had a nuchal translucency measurement between 3.5 and less than 5 mm, 68 (21.5%) had a measurement between 5 and less than 6.5 mm, and 100 (31.6%) had a nuchal translucency of 6.5 mm or more. The characteristics of the study population are summarized in Table 1. The flow chart illustrating study inclusion, pregnancy follow‐up, and outcomes is detailed in Figure 1.

**TABLE 1:** Population characteristics, (*n* = 316).

Characteristics	*N*	Total
Age (years. Mean ± standard deviation)	316	31.6 ± 6.4
BMI (kg/m^2^. Mean ± standard deviation)	316	25.8 ± 6.9
Smoking during pregnancy, *n* (%)	264	29 (11.0)
Alcohol consumption during pregnancy, *n* (%)	265	4 (1.5)
Parity, *n* (%)	316	
Nulliparous		109 (34.5)
Primiparous		112 (35.4)
Multiparous		95 (30.1)
Relevant maternal medical history, *n* (%)	316	19 (6.0)
History of early pregnancy loss, *n* (%)	316	78 (24.7)
History of increased nuchal translucency, *n* (%)	316	8 (2.5)
History of stillbirth, *n* (%)	316	4 (1.3)
History of fetal pathology, *n* (%)	316	13 (4.1)
History of familial congenital disorder, *n* (%)	316	16 (5.1)
Nuchal translucency, *n* (%)	316	
≥ 3.5 mm to < 5 mm		148 (46.8)
≥ 5 mm to < 6.5 mm		68 (21.5)
≥ 6.5 mm		100 (31.6)
Hydrops fetalis, *n* (%)	261	69 (26.4)
Fetal weight estimation		
Early second trimester morphology ultrasound^a^ percentile (median [interquartile range])	47	17 ± 41
Second‐trimester ultrasound percentile (median [interquartile range])	38	46 ± 56
Third‐trimester ultrasound percentile (median [interquartile range])	20	38 ± 52
Ultrasound fetal sex, *n* (%)	125	
Male		74 (59.2)
Female		51 (40.8)

^a^ Early second trimester: corresponding to approximately 16–18 weeks of gestation.

Pregnancy management strategies are summarized in Table 2. Among the 248 invasive prenatal procedures with available timing data, 180 (72.6%) were performed during the first trimester and 63 (25.4%) during the early second trimester. Genetic sampling predominantly involved chorionic villus sampling (180/316, 57.0%), performed rather than amniocentesis. TOP was also frequently initiated at these early stages: 69.4% (125/180) in the first trimester and 18.3% (33/180) in the early second trimester. Among the five couples who underwent invasive testing in the second trimester or later, four had not been referred earlier in pregnancy, and in one case, a structural anomaly detected at the second‐trimester ultrasound prompted the indication for genetic sampling. Stillbirths predominantly occurred in the later stages of pregnancy, with six cases in the second (50.0%) and four in the third trimester (33.3%) of gestation.

**TABLE 2:** Pregnancy management and explorations, (*n* = 316).

Characteristics	*N*	Total
Gestational age at invasive genetic sampling	248	
First trimester, *n* (%)		180 (72.6)
Early second trimester^a^, *n* (%)		63 (25.4)
Second trimester, *n* (%)		3 (1.2)
Third trimester, *n* (%)		2 (0.8)
Mean ± standard deviation (weeks of gestation)		14.1 ± 2.4
Type of invasive genetic sampling, *n* (%)	316	
Chorionic villus sampling		180 (57.0)
Amniocentesis		68 (21.5)
Not desired		68 (21.5)
Gestational age at termination of pregnancy	180	
First trimester, *n* (%)		125 (69.4)
Early second trimester^a^, *n* (%)		33 (18.3)
Second trimester, *n* (%)		20 (11.1)
Third trimester, *n* (%)		2 (1.1)
Mean ± standard deviation (weeks of gestation)		15.7 ± 3.3
Last examination before requesting termination of pregnancy, *n* (%)	176	
Genetic examination		123 (69.9)
Morphological ultrasound		53 (30.1)
Gestational age at stillbirth	12	
First trimester, *n* (%)		2 (16.7)
Early second trimester^a^, *n* (%)		0 (0.0)
Second trimester, *n* (%)		6 (50.0)
Third trimester, *n* (%)		4 (33.3)
Mean ± standard deviation (weeks of gestation)		20.3 ± 8.3

^a^ Early second trimester: corresponding to approximately 16–18 weeks of gestation.

Pregnancy outcomes varied markedly according to NT thickness (Table 3). Overall, 91/316 pregnancies (28.8%) resulted in a live birth without detected pathology, whereas 180/316 (57.0%) resulted in termination of pregnancy. The proportion of live births without pathology decreased from 43.9% in the 3.5–< 5.0 mm group to 25.0% in the 5.0–< 6.5 mm group and 9.0% in the ≥ 6.5 mm group. Conversely, termination of pregnancy increased from 42.6% to 60.3% and 76.0%, respectively. Stillbirths occurred predominantly in the ≥ 6.5 mm group, which accounted for 9 of the 12 cases.

**TABLE 3:** Pregnancy outcomes based on nuchal translucency measurement at first‐trimester ultrasound.

Outcome	Nuchal translucency, (nT) measurement			
	Overall *n* = 316	3.5 ≤ NT < 5 mm *n* = 148	5 ≤ NT < 6.5 mm *n* = 68	NT ≥ 6.5 mm *n* = 100
Live birth without pathology, *n* (%)	91 (28.8)	65 (43.9)	17 (25.0)	9 (9.0)
Termination of pregnancy, *n* (%)	180 (57.0)	63 (42.6)	41 (60.3)	76 (76.0)
Stillbirth, *n* (%)	12 (3.8)	2 (1.4)	1 (1.5)	9 (9.0)
Postnatal death, *n* (%)	8 (2.5)	3 (2.0)	4 (5.9)	1 (1.0)
Live birth with pathology, *n* (%)	25 (7.9)	15 (10.1)	5 (7.4)	5 (5.0)
Pregnancies still ongoing at ≥ 14 weeks' gestation, *n* (%)	238 (75.3)	126 (85.1)	50 (73.5)	62 (62.0)
Pregnancies still ongoing at ≥ 18 weeks' gestation, *n* (%)	157 (49.7)	95 (64.2)	32 (47.1)	30 (30.0)
Pregnancies still ongoing at ≥ 22 weeks' gestation, *n* (%)	135 (42.7)	84 (56.8)	29 (42.6)	22 (22.0)
Pregnancies still ongoing at ≥ 32 weeks' gestation, *n* (%)	113 (35.8)	74 (50.0)	24 (35.3)	15 (15.0)
Pregnancies still ongoing at ≥ 37 weeks' gestation, *n* (%)	103 (32.6)	71 (48.0)	20 (29.4)	12 (12.0)

**FIGURE 2:**
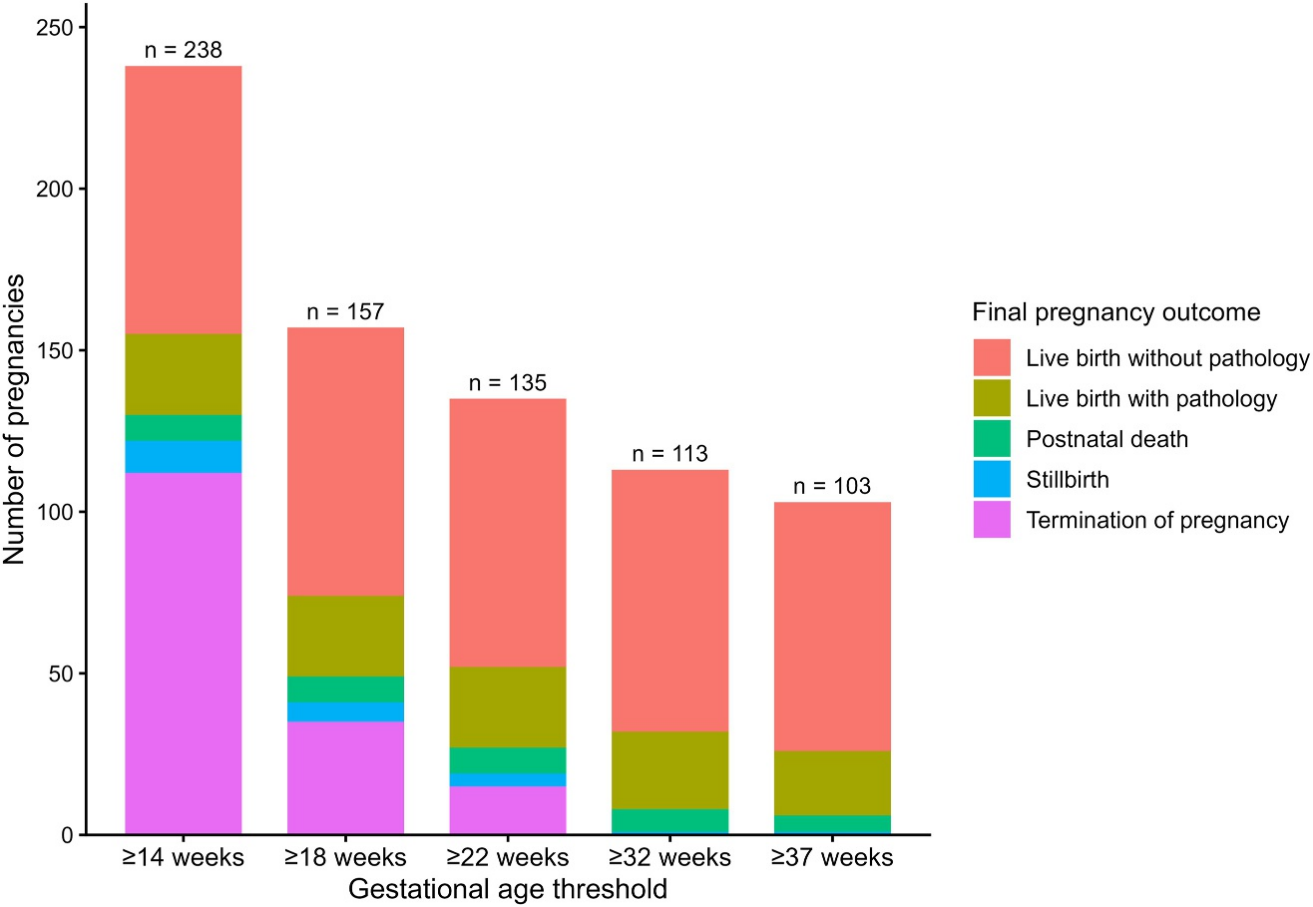
Final pregnancy outcomes according to gestational age among pregnancies still ongoing at each selected threshold.

Among pregnancies still ongoing at successive gestational age thresholds, the proportion resulting in a live birth without detected pathology increased from 34.9% at ≥ 14 weeks to 74.8% at ≥ 37 weeks, whereas termination of pregnancy declined from 47.1% to 0% by ≥ 32 weeks (Figure 2 and Supporting Information S1: Table S1). Nevertheless, 20/103 pregnancies (19.4%) reaching ≥ 37 weeks resulted in a live birth with pathology.

Parental decision‐making showed a marked gradient across the NT categories (Table 4). Termination of pregnancy without prior invasive prenatal testing occurred in 61/316 cases (19.3%), increasing from 11/148 (7.4%) in the 3.5–< 5.0 mm group to 15/68 (22.1%) in the 5.0–< 6.5 mm group and 35/100 (35.0%) in the ≥ 6.5 mm group. Conversely, invasive prenatal testing was performed in 126/148 (85.1%), 58/68 (85.3%), and 64/100 (64.0%) pregnancies, respectively. NIPT alone was chosen in only four pregnancies: two in the 3.5–< 5.0 mm group and two in the ≥ 6.5 mm group.

**TABLE 4:** Parental decision‐making and pregnancy continuation according to nuchal translucency thickness.

	Overall *N* = 316	3.5 ≤ NT < 5 mm *N* = 148	5 ≤ NT < 6.5 mm *N* = 68	NT ≥ 6.5 mm *N* = 100
Termination of pregnancy before invasive testing, *n* (%)	61 (19.3)	11 (7.4)	15 (22.1)	35 (35.0)
Invasive prenatal testing, *n* (%)	248 (78.5)	126 (85.1)	58 (85.3)	64 (64.0)
NIPT alone, *n* (%)	4 (1.3)	2 (1.4)	0 (0.0)	2 (2.0)
Pregnancy continuation after invasive testing, *n*/*N* (%)	116/248 (46.8)	72/126 (57.1)	26/58 (44.8)	18/64 (28.1)
Pregnancy continuation despite an abnormal invasive genetic test result, *n/N* (%)	12/137 (8.8)	5/52 (9.6)	3/33 (9.1)	4/52 (7.7)
Structural ultrasound examination, *n* (%)	111 (35.1)	72 (48.6)	22 (32.4)	17 (17.0)
Pregnancy continuation after structural ultrasound assessment, *n/N* (%)	90/111 (81.1)	55/72 (76.4)	21/22 (95.5)	14/17 (82.4)
Pregnancy continuation despite abnormal structural ultrasound findings, *n/N* (%)	17/29 (58.6)	9/15 (60.0)	3/7 (42.9)	5/7 (71.4)

Abbreviation: NIPT: non‐invasive prenatal testing.

Following invasive prenatal testing, pregnancy was continued in 116/248 cases (46.8%). Continuation rates decreased with increasing NT thickness, from 72/126 (57.1%) in the 3.5–< 5.0 mm group to 26/58 (44.8%) in the 5.0–< 6.5 mm group and 18/64 (28.1%) in the ≥ 6.5 mm group. Among the 137 pregnancies with an abnormal invasive genetic test result, 12 (8.8%) were continued, including 5/52 (9.6%), 3/33 (9.1%), and 4/52 (7.7%) across the three NT categories, respectively.

Structural ultrasound assessment was performed in 111/316 pregnancies (35.1%), including 72/148 (48.6%) in the 3.5–< 5.0 mm group, 22/68 (32.4%) in the 5.0–< 6.5 mm group, and 17/100 (17.0%) in the ≥ 6.5 mm group. Pregnancy was continued after structural ultrasound assessment in 90/111 cases (81.1%), including 55/72 (76.4%), 21/22 (95.5%), and 14/17 (82.4%) across the three NT categories, respectively. Among the 29 pregnancies with at least one abnormal structural ultrasound finding, 17 (58.6%) were continued and 12 (41.4%) were terminated. Continuation despite abnormal structural findings occurred in 9/15 (60.0%), 3/7 (42.9%), and 5/7 (71.4%) pregnancies across the three NT categories, respectively.

Among the 51 pregnancies that continued after both normal prenatal genetic testing and normal structural ultrasound assessment, 42 (82.4%) resulted in a live birth without detected pathology at the last available follow‐up. This proportion was 31/34 (91.2%) in the 3.5–< 5.0 mm group, 8/9 (88.9%) in the 5.0–< 6.5 mm group, and 3/8 (37.5%) in the ≥ 6.5 mm group.

Among the 22 requests for termination of pregnancy in the second or third trimester, eight (36.4%) involved cases in which the fetal pathology was already known by the end of the first trimester (either through ultrasound or genetic testing) but the parents required additional time for decision‐making. In eight other cases (36.4%), invasive sampling was performed later than average, around 18 weeks of gestation. The remaining six (27.3%) had undergone first‐trimester sampling, but abnormal results were only revealed after CMA or exome sequencing, necessitating a longer diagnostic and counseling process.

The documented indications for termination of pregnancy are presented in Table 5. Overall, abnormal genetic findings were the leading indication, accounting for 117/180 terminations (65.0%), with similar proportions across gestational age categories. Among terminations following an abnormal prenatal genetic result, the genetic finding itself was the primary documented indication in 115/125 cases (92.0%). In contrast, among the 12 terminations following abnormal structural ultrasound findings, the decision was most frequently attributed to concerns regarding the child's prognosis (5/12, 41.7%), followed by the structural abnormality itself (4/12, 33.3%) and associated genetic findings (3/12, 25.0%).

**TABLE 5:** Motivations for requests for termination of pregnancy.

	All terminations				Terminations after an abnormal genetic result				Terminations after abnormal structural ultrasound findings		
	Overall *N* = 180	First trimester *N* = 125	Early 2nd trimester *N* = 33	2nd and 3rd trimester *N* = 22	Overall *N* = 125	First trimester *N* = 84	Early 2nd trimester *N* = 25	2nd and 3rd trimester *N* = 16	Overall *N* = 12	Early 2nd trimester *N* = 2	2nd and 3rd trimester *N* = 10
Genetic test results, *n* (%)	117 (65.0)	81 (64.8)	22 (66.7)	14 (63.6)	115 (92.0)	79 (94.1)	22 (88.0)	14 (87.5)	3 (25.0)	0 (0.0)	3 (30.0)
Morphological examination results, *n* (%)	38 (21.1)	28 (22.4)	7 (21.2)	3 (13.6)	5 (4.0)	3 (3.6)	2 (8.0)	0 (0.0)	4 (33.3)	1 (50.0)	3 (30.0)
Concerns about child prognosis, *n* (%)	20 (11.1)	12 (9.6)	3 (9.1)	5 (22.7)	4 (3.2)	1 (1.2)	1 (4.0)	2 (12.5)	5 (41.7)	1 (50.0)	4 (40.0)
Other, *n* (%)	2 (1.1)	1 (0.8)	1 (3.0)	0 (0.0)	0 (0.0)	0 (0.0)	0 (0.0)	0 (0.0)	0 (0.0)	0 (0.0)	0 (0.0)
Missing, *n* (%)	3 (1.7)	3 (2.4)	0 (0.0)	0 (0.0)	1 (0.8)	1 (1.2)	0 (0.0)	0 (0.0)	0 (0.0)	0 (0.0)	0 (0.0)

Detailed structural and genetic findings are presented in Supporting Information S1: Tables S2–S5. Among the 111 pregnancies that underwent structural ultrasound assessment, 82 (73.9%) had no structural abnormality detected, while cardiac malformations were the most frequent findings (15/111, 13.5%); regression of the increased NT was observed in 77/111 cases (69.4%). Genetic testing yielded an abnormal result in 142/253 pregnancies (56.1%), predominantly aneuploidies (115/253, 45.5%). The causative abnormality was most often first identified by rapid FISH (91/142, 64.1%), followed by chromosome analysis, CMA, and exome sequencing. The distribution of genetic diagnoses and the diagnostic techniques according to malformation type are detailed in Supporting Information S1: Tables S3–S5.

Among the 25 live births with pathology, 14 had a structural abnormality identified prenatally and two had a known genetic diagnosis; six had no structural ultrasound assessment and four declined invasive testing. Among the 20 born at ≥ 37 weeks, 10 had a known structural abnormality, five had no structural ultrasound assessment, and three declined invasive testing.

## 4 Discussion

This retrospective cohort study delineates the diagnostic and care trajectories, parental decision‐making, and pregnancy outcomes of pregnancies with increased NT identified in the first trimester. Most cases underwent early genetic investigations, while TOP was also frequent and usually occurred before 18 weeks of gestation. Parental decisions varied markedly according to NT thickness. Termination of pregnancy without prior invasive testing became more frequent as NT increased, whereas pregnancy continuation after invasive testing decreased. This pattern was most pronounced in the ≥ 6.5 mm group, suggesting that NT thickness itself may influence parental perceptions of prognosis before definitive diagnostic results are available. However, parental decisions are also shaped by the content and framing of counseling provided by healthcare professionals, which may itself vary according to NT thickness and perceived prognosis. Because these interactions were not systematically documented, their contribution could not be assessed. [13, 14] Abnormal genetic test results were the most frequently documented factor associated with parental decision‐making, particularly regarding termination of pregnancy. Nevertheless, some pregnancies were continued despite abnormal genetic or structural ultrasound findings, highlighting the individualized nature of parental decision‐making. Decisions may depend on the type and expected severity of the condition, prognostic uncertainty, gestational age at diagnosis, and individual parental values. The relatively high continuation rate after abnormal structural ultrasound findings should therefore not be interpreted as indicating limited clinical significance of these abnormalities. After normal prenatal genetic testing and normal structural ultrasound assessment, most continued pregnancies resulted in live birth without detected pathology, although this favorable outcome was substantially less frequent when NT was ≥ 6.5 mm.

The demographic characteristics of the cohort were broadly consistent with those previously reported. A history of miscarriage was reported in 24.7% of pregnancies, higher than approximately 15% reported in recent literature [15], which may partly reflect the slightly older maternal age in our cohort.

We chose to include cystic hygromas within the NT category because of the lack of consistent definitions in clinical practice, reflecting real‐world diagnostic variability. Similarly, we did not further stratify cases of live births with pathology by severity, acknowledging the subjectivity and ethical complexities in defining what constitutes a “severe” versus “mild” condition in a neutral and universally acceptable way.

The timing of referrals and evaluations closely mirrored usual clinical pathways, with most cases undergoing workup during the first or early second trimester. Some diagnostic delays occurred due to referral wait times, explaining why a significant proportion of tests occurred after 18 weeks. Later, invasive testing was mainly related to delayed referral or to abnormalities identified during subsequent ultrasound assessment. These findings underline the importance of timely access to diagnostic testing and counseling throughout the decision‐making process. Later TOP did not necessarily reflect delayed parental decision‐making, as some couples required additional time after an early diagnosis, whereas others received definitive genetic or structural information only later in pregnancy.

However, decisions were not solely based on objective findings. A diagnosis of markedly increased NT is likely to be psychologically burdensome for expectant parents, particularly because of prognostic uncertainty, waiting times for results, and concerns about severe disability [16]. These factors may have influenced decisions regarding pregnancy continuation or termination; however, our retrospective design did not allow us to evaluate the extent to which psychological distress contributed to such choices.

Approximately 80% of patients opted for genetic testing, although termination without prior invasive testing became increasingly frequent with greater NT thickness. NIPT alone may be insufficient after increased NT, as approximately 20% of the chromosomal abnormalities identified in our cohort would not have been detected by standard screening for common trisomies. [17] CMA identified approximately 10% of additional diagnoses after normal rapid aneuploidy testing or chromosome analysis, compared with about 5% in previous meta‐analyses, although differences in testing strategies may partly explain this discrepancy. [18] Exome sequencing provided additional diagnoses, including RASopathies, supporting its consideration in selected cases after normal chromosome analysis or CMA. [5, 19, 20].

Ultrasound monitoring also plays an important role in clinical management. The 18‐week scan appeared to be an important step in the diagnostic pathway, allowing early detection of structural anomalies and contributing to subsequent counseling and decision‐making. Our findings are consistent with current recommendations advocating mid‐trimester ultrasound after increased NT [21]. The distribution of ultrasound‐detected anomalies in our cohort was consistent with existing literature [22], with cardiac defects being the most frequently observed, followed by syndromic or polymalformative presentations; limb anomalies were uncommon. These findings support systematic fetal cardiac assessment after increased NT.

Despite the high prevalence of anomalies, not all increased NT cases have poor prognoses. Although only 28.8% of the overall cohort resulted in live birth without detected pathology, this proportion reached 82.4% among pregnancies continued after normal prenatal genetic testing and normal structural ultrasound assessment. However, this favorable outcome was observed in only 3/8 pregnancies with NT ≥ 6.5 mm, indicating that substantial residual risk persists despite reassuring prenatal investigations in this subgroup. This finding supports cautious counseling, as reassuring prenatal investigations substantially improve prognosis but do not eliminate residual risk, particularly when NT is ≥ 6.5 mm. These conditional estimates may be more informative for counseling than outcomes calculated from the entire cohort, because they reflect prognosis after successive reassuring investigations. Nevertheless, the small number of pregnancies with NT ≥ 6.5 mm reaching this stage requires cautious interpretation. The gestational age‐conditional analysis confirms a well‐established pattern: the likelihood of a live birth without detected pathology improves markedly with advancing gestation, irrespective of initial NT thickness. This is consistent with the findings of Gadsbøll et al. [23], and with the landmark data from Souka et al. [1]. These findings reinforce the importance of incorporating gestational age at assessment into prenatal counseling, particularly when discussing residual risk after reassuring genetic and ultrasound results. The relatively high proportion of live births with pathology among pregnancies reaching ≥ 37 weeks partly reflects the conditional nature of this subgroup: earlier terminations were excluded, whereas pregnancies continued despite known abnormalities remaining. Moreover, pathology included any diagnosed condition, irrespective of severity.

The 12‐year study period and the inclusion of all major pregnancy outcomes provide a detailed overview of care trajectories in routine clinical practice. The single‐center setting helped maintain consistent protocols, high‐quality imaging (validated by Herman score and certified sonographers), and consistent multidisciplinary assessment, including genetics and pediatric cardiology. A further strength is the detailed reconstruction of sequential parental decisions according to NT thickness and the results available at each stage of the diagnostic pathway.

However, the retrospective design may introduce selection bias. As a single‐center study, our findings may not fully generalize to other settings. Nonetheless, our center receives referrals from a wide region, which likely reduces this limitation. One methodological limitation lies in the potential underrepresentation of cases not referred for specialized consultation. In our country, elective termination is legally permitted up to 16 weeks of gestation, which raises the possibility that some patients with markedly increased NT opted for early termination without ever being seen at our center. This may have biased our sample toward more complex, ambiguous, or later‐presented cases. Additionally, a substantial proportion of pregnancies did not undergo complete diagnostic workup, particularly structural ultrasound assessment. However, 94.6% of pregnancies without structural ultrasound assessment already had undergone termination before 18 weeks of gestation, reflecting the clinical context. This limits the generalizability of ultrasound‐related findings but is less likely to affect the interpretation of genetic diagnostic trajectories. The NT stratification used in this study reflects our institutional practice and may limit direct comparability with studies using alternative cutoffs; however, the observed outcome distributions across strata remain broadly consistent with published series. Finally, the absence of documented late‐onset pathology cannot exclude conditions diagnosed outside regional pediatric records or after the available follow‐up period.

Our data suggest that prenatal counseling may benefit from a stepwise framework integrating NT thickness, sequential genetic testing, structural ultrasound findings, and the evolving prognosis throughout pregnancy.

## 5 Conclusion

First‐trimester increased NT is associated with markedly different diagnostic pathways, parental decisions, and pregnancy outcomes according to NT thickness. Termination without prior invasive testing became more frequent as NT increased, whereas pregnancy continuation after invasive testing decreased. Although most pregnancies continued after normal prenatal genetic testing and structural ultrasound assessment resulted in a live birth without detected pathology, this favorable outcome remained substantially less frequent when NT was ≥ 6.5 mm. These findings may support a more individualized prenatal counseling by integrating NT thickness, successive diagnostic results, and the residual risk of an adverse outcome.

Future studies should include prospective multicenter cohorts to further characterize care pathways and assess the impact of specialized counseling on parental distress and decision‐making.

## Funding

The authors have nothing to report.

## Ethics Statement

The authors state that they have adhered to the ethical principles outlined in the Declaration of Helsinki. This study was approved by our local ethics committee (IRB No. 2024‐OBS‐1209)12.

## Consent

The authors have nothing to report.

## Conflicts of Interest

The authors declare no conflicts of interest.

## Supporting information


Supporting Information S1

## Data Availability

The data that support the findings of this study are available on request from the corresponding author. The data are not publicly available due to privacy or ethical restrictions.
