# Supplementary material for: Parental Decision‐Making and Pregnancy Outcomes After Increased First‐Trimester Nuchal Translucency: A 12‐Year Cohort
Source: Prenat Diagn. 2026 Jul 14;46(10):1520–8. doi: 10.1002/pd.70224 (PMC13571791; doi:10.1002/pd.70224)
Supplement: Supplementary file 1 [file PD-46-1520-s001.docx]

**Supplementary Table 1. Distribution of pregnancy outcomes among pregnancies still ongoing at selected gestational age thresholds**

| Outcome | ≥14 weeks  n = 238 | ≥18 weeks  n = 157 | ≥22 weeks  n = 135 | ≥32 weeks  n =113 | ≥37 weeks  n = 103 |
| --- | --- | --- | --- | --- | --- |
| Live birth without pathology, n (%) | 83 (34.9) | 83 (52.9) | 83 (61.5) | 81 (71.7) | 77 (74.8) |
| Termination of Pregnancy, n (%) | 112 (47.1) | 35 (22.3) | 15 (11.1) | 0 (0.0) | 0 (0.0) |
| Stillbirth, n (%) | 10 (4.2) | 6 (3.8) | 4 (3.0) | 1 (0.9) | 1 (1.0) |
| Postnatal death, n (%) | 8 (3.4) | 8 (5.1) | 8 (5.9) | 7 (6.2) | 5 (4.9) |
| Live birth with pathology, n (%) | 25 (10.5) | 25 (15.9) | 25 (18.5) | 24 (21.2) | 20 (19.4) |

# Supplementary Table 2. Findings from morphological ultrasounds

| Results | Overall  n = 111 | 3.5 ≤ NT < 5mm  n = 72 | 5 ≤ NT < 6.5mm  n = 22 | NT ≥ 6.5mm  n = 17 |
| --- | --- | --- | --- | --- |
| Normal, n (%) | 82 (73.9) | 57 (79.2) | 15 (68.2) | 10 (58.8) |
| Cardiac malformation, n (%) | 15 (13.5) | 9 (12.5) | 3 (13.6) | 3 (17.6) |
| *Ventricular septal defect* | *4 (3.6)* | *3 (4.2)* | *0 (0.0)* | *1 (5.9)* |
| *Hypoplastic left heart syndrome* | *3 (2.7)* | *1 (1.4)* | *2 (9.1)* | *0 (0.0)* |
| *Tetralogy of Fallot* | *2 (1.8)* | *1 (1.4)* | *1 (4.5)* | *0 (0.0)* |
| *Cardiac chamber asymmetry* | *1 (0.9)* | *1 (1.4)* | *0 (0.0)* | *0 (0.0)* |
| *Complex congenital heart disease* | *1 (0.9)* | *0 (0.0)* | *0 (0.0)* | *1 (5.9)* |
| *Great vessel asymmetry* | *1 (0.9)* | *0 (0.0)* | *0 (0.0)* | *1 (5.9)* |
| *Hypertrophic cardiomyopathy* | *1 (0.9)* | *1 (1.4)* | *0 (0.0)* | *0 (0.0)* |
| *Transposition of the great vessels* | *1 (0.9)* | *1 (1.4)* | *0 (0.0)* | *0 (0.0)* |
| *Unspecified* | *1 (0.9)* | *1 (1.4)* | *0 (0.0)* | *0 (0.0)* |
| Kidney and urinary tract malformation, n (%) | 8 (7.2) | 5 (6.9) | 2 (9.1) | 1 (5.9) |
| *Bilateral pyelectasis* | *3 (2.7)* | *2 (2.8)* | *0 (0.0)* | *1 (5.9)* |
| *Unilateral pyelectasis* | *2 (1.8)* | *2 (2.8)* | *0 (0.0)* | *0 (0.0)* |
| *Megacystis* | *1 (0.9)* | *1 (1.4)* | *0 (0.0)* | *0 (0.0)* |
| *Unilateral ectopic kidney* | *1 (0.9)* | *0 (0.0)* | *1 (4.5)* | *0 (0.0)* |
| *Unilateral multicystic dysplastic kidney* | *1 (0.9)* | *0 (0.0)* | *1 (4.5)* | *0 (0.0)* |
| Hand and foot malformation, n (%) | 3 (2.7) | 2 (2.8) | 0 (0.0) | 1 (5.9) |
| Congenital bone disease, n (%) | 3 (2.7) | 2 (2.8) | 0 (0.0) | 1 (5.9) |
| Polymalformative syndrome, n (%) | 7 (6.3) | 0 (0.0) | 4 (18.2) | 3 (17.6) |
| Other malformations, n (%) | 6 (5.4) | 1 (1.4) | 2 (9.1) | 3 (17.6) |
| *Diaphragmatic hernia* | *1 (0.9)* | *0 (0.0)* | *1 (4.5)* | *0 (0.0)* |
| *Enlarged sylvian fissure* | *1 (0.9)* | *0 (0.0)* | *0 (0.0)* | *1 (5.9)* |
| *Esophageal atresia* | *1 (0.9)* | *1 (1.4)* | *0 (0.0)* | *0 (0.0)* |
| *Exencephaly* | *1 (0.9)* | *0 (0.0)* | *1 (4.5)* | *0 (0.0)* |
| *Late hydrops fetalis* | *2 (1.8)* | *0 (0.0)* | *0 (0.0)* | *2 (11.8)* |
| Unilateral *hydrothorax* | *1 (0.9)* | *0 (0.0)* | *0 (0.0)* | *1 (5.9)* |
| Regression of increased nuchal translucency, n (%) | 77 (69.4) | 51 (70.8) | 19 (86.4) | 7 (41.2) |

*Fetuses presenting with multiple findings may be included in more than one category*

*NT : nuchal translucency*

# Supplementary Table 3. First genetic test identifying the causative anomaly

| Genetic test | Overall  n = 142 | 3.5 ≤ NT < 5mm  n = 57 | 5 ≤ NT < 6.5mm  n = 33 | NT ≥ 6.5mm  n = 52 |
| --- | --- | --- | --- | --- |
| Rapid Fluorescence In Situ Hybridization, n (%) | 91 (64.1) | 35 (61.4) | 24 (72.7) | 32 (61.5) |
| Chromosome analysis, n (%) | 22 (15.5) | 7 (12.3) | 4 (12.1) | 11 (21.2) |
| Chromosomal Microarray Analysis, n (%) | 13 (9.2) | 6 (10.5) | 2 (6.1) | 5 (9.6) |
| Prenatal exome sequencing, n (%) | 4 (2.8) | 3 (5.3) | 0 (0.0) | 1 (1.9) |
| Postnatal exome sequencing, n (%) | 12 (8.5) | 6 (10.5) | 3 (9.1) | 3 (5.8) |

# Supplementary Table 4. Identified genetic etiologies

| Etiology | Overall  n = 253 | 3.5 ≤ NT < 5mm  n = 130 | 5 ≤ NT < 6.5mm  n = 59 | NT ≥ 6.5mm  n = 64 |
| --- | --- | --- | --- | --- |
| Abnormal findings, n (%) | 142 (56.1) | 57 (43.8) | 33 (55.9) | 52 (81.3) |
| *Aneuploidy* | *115 (45.5)* | *42 (32.3)* | *28 (47.5)* | *45 (70.3)* |
| *Trisomy 21 (Down syndrome)* | *64 (25.3)* | *29 (22.3)* | *16 (27.1)* | *19 (29.7)* |
| *Monosomy X (Turner syndrome)* | *24 (9.5)* | *1 (0.8)* | *2 (3.4)* | *21 (32.8)* |
| *Trisomy 18 (Edwards syndrome)* | *21 (8.3)* | *9 (6.9)* | *7 (11.9)* | *5 (7.8)* |
| *Trisomy 13 (Patau syndrome)* | *4 (1.6)* | *1 (0.8)* | *3 (5.1)* | *0 (0.0)* |
| *Trisomy 22* | *2 (0.8)* | *2 (1.5)* | *0 (0.0)* | *0 (0.0)* |
| *CNV* | *12 (4.7)* | *7 (5.4)* | *2 (3.4)* | *3 (4.7)* |
| *DiGeorge (22q11 deletion) syndrome* | *4 (1.6)* | *1 (0.8)* | *2 (3.4)* | *1 (1.6)* |
| *Williams-Beuren syndrome* | *2 (0.8)* | *2 (1.5)* | *0 (0.0)* | *0 (0.0)* |
| *16p11.2 deletion* | *1 (0.4)* | *1 (0.8)* | *0 (0.0)* | *0 (0.0)* |
| *18p10 deletion* | *1 (0.4)* | *1 (0.8)* | *0 (0.0)* | *0 (0.0)* |
| *4q and 5q35.3 deletion* | *1 (0.4)* | *1 (0.8)* | *0 (0.0)* | *0 (0.0)* |
| *6q23 deletion* | *1 (0.4)* | *0 (0.0)* | *0 (0.0)* | *1 (1.6)* |
| *8p23.1 deletion* | *1 (0.4)* | *0 (0.0)* | *0 (0.0)* | *1 (1.6)* |
| *9q34.2qter duplication and 15q26.2qter deletion (unbalanced translocation)* | *1 (0.4)* | *1 (0.8)* | *0 (0.0)* | *0 (0.0)* |
| *SNV* | *15 (5.9)* | *8 (6.2)* | *3 (5.1)* | *4 (6.3)* |
| *Noonan syndrome* | *5 (2.0)* | *3 (2.3)* | *0 (0.0)* | *2 (3.1)* |
| *RASopathy (excluding Noonan syndrome)* | *3 (1.2)* | *1 (0.8)* | *0 (0.0)* | *2 (3.1)* |
| *Cornelia de Lange syndrome* | *1 (0.4)* | *0 (0.0)* | *1 (1.7)* | *0 (0.0)* |
| *Kallmann syndrome* | *1 (0.4)* | *0 (0.0)* | *1 (1.7)* | *0 (0.0)* |
| *Neurofibromatosis type I* | *1 (0.4)* | *0 (0.0)* | *1 (1.7)* | *0 (0.0)* |
| *Heterozygous COL4A3 variant* | *1 (0.4)* | *1 (0.8)* | *0 (0.0)* | *0 (0.0)* |
| *Kabuki syndrome* | *1 (0.4)* | *1 (0.8)* | *0 (0.0)* | *0 (0.0)* |
| *Multiple pterygium syndrome* | *1 (0.4)* | *1 (0.8)* | *0 (0.0)* | *0 (0.0)* |
| *Unspecified exome variant* | *1 (0.4)* | *1 (0.8)* | *0 (0.0)* | *0 (0.0)* |
| Normal, n (%) | 111 (43.9) | 73 (56.2) | 26 (44.1) | 12 (18.8) |

# *CMA : Chromosomal Microarray Analysis, CNV : Copy Number Variant, FISH : Fluorescence In Situ Hybridization, SNV : Single Nucleotide Variant*

# Supplementary Table 5. Distribution of the first genetic test identifying the causative anomaly by type of congenital malformation

| Category, n (%) | Normal | Test | | |
| --- | --- | --- | --- | --- |
|  |  | FISH or Chromosome analysis | CMA | Exome sequencing |
| Cardiac malformations | 9 (60.0) | 1 (6.7) | 1 (6.7) | 4 (26.7) |
| Kidney and urinary tract malformations | 3 (37.5) | 1 (12.5) | 0 (0.0) | 4 (50.0) |
| Hand and foot malformations | 1 (33.3) | 1 (33.3) | 1 (33.3) | 0 (0.0) |
| Congenital bone disease | 1 (33.3) | 0 (0.0) | 0 (0.0) | 2 (66.7) |
| Polymalformative syndromes | 3 (42.9) | 3 (42.9) | 0 (0.0) | 1 (14.3) |
| Other malformations | 2 (28.6) | 2 (28.6) | 1 (14.3) | 2 (28.6) |
| Nuchal translucency persistence | 22 (59.5) | 3 (8.1) | 5 (13.5) | 7 (18.9) |

*CMA : Chromosomal Microarray Analysis, FISH : Fluorescence In Situ Hybridization*
